# Improving COVID-19 Vaccination Uptake in Service Users Admitted to an Acute Inpatient Psychiatric Ward

**DOI:** 10.1192/bjo.2022.445

**Published:** 2022-06-20

**Authors:** Miranda Holliday, Lisanne Stock, Bhina Patel, Fidaa Natour, Victor Cohn, Mercedes Chavarri

**Affiliations:** Barnet, Enfield and Haringey Mental Health NHS Trust, London, United Kingdom

## Abstract

**Aims:**

It is well known that individuals suffering from mental illness have more comorbidities and lower life expectancies compared to the general population. It is unsurprising therefore, that these individuals are more vulnerable to both contracting COVID-19, and developing severe illness if infected. When patients are admitted to a psychiatric inpatient unit this offers an invaluable opportunity to ensure that unvaccinated patients are identified, and if consenting, are supported to receive whichever dose of the vaccine they require. We undertook an audit to examine the proportion of patients admitted who had not received their first, second or third dose of the COVID-19 vaccination. Reviewed in the context of gender, age, ethnicity, legal status, mental health diagnoses and additional comorbidities, in order to determine any trends that might assist in improving uptake. We then repeated the audit aiming to offer the appropriate COVID-19 vaccination to every newly admitted unvaccinated patient. If refused, to then council reluctant patients, providing simple, understandable vaccine information, and to re-offer vaccination.

**Methods:**

The audit took place on a mixed adult psychiatric inpatient ward in London.

The first cycle of the audit was completed retrospectively. Data were collected from the electronic notes of new admissions from November and December 2021 (total 41). This included information on COVID-19 vaccination status, and documentation of vaccines offered and administered during admission. Additional information was also compiled to calculate risk stratification scores.

Subsequently, we repeated the audit cycle for admissions in January and February 2022 (on-going). However, this time with the aim that all patients have their COVID-19 vaccination status documented promptly, and that their next vaccination is offered/administered during admission if required.

**Results:**

Results from the initial audit cycle showed 33/41 patients had not received a full set of COVID-19 vaccinations (or no vaccination record found). Only 6/33 unvaccinated patients were offered the next vaccination during admission, and 3/33 actually received one. 21/33 patients without a full set of vaccinations were BAME (Black, Asian and minority ethnic).

Initial results from the second cycle showed an improvement in the number of patients offered the vaccine. 5/10 unvaccinated patients were offered vaccines in January, however data collection is ongoing.

**Conclusion:**

Although our data set is not yet complete, initial results show that a simple intervention such as early identification of unvaccinated patients on admission, can act as a prompt to clinicians to ensure vaccines are offered. Thereby, increasing vaccine compliance in this vulnerable patient group.

